# Experiences and projections for the future of research, training and other academic activities: Will it be the same?

**DOI:** 10.1192/j.eurpsy.2021.47

**Published:** 2021-08-13

**Authors:** P. Mohr

**Affiliations:** 1 Psychiatric Clinic, 3rd Faculty of Medicine, Charles University, Praha, Czech Republic; 2 Clinical Dept., National Institute of Mental Health, Klecany, Klecany, Czech Republic

## Abstract

The global SARS-CoV-2 pandemic with subsequently imposed restrictions and lockdowns also radically disrupted academic life. Many research projects involving recruitment of human subjects were abruptly put on hold, educational activities have moved into online trainings, scientific meetings have been transformed into virtual events. Social distancing does not restrict only everyday human contact but also limits direct exchange of clinical, educational, and research experiences, professional and academic networking, sharing ideas. Besides all the drawbacks, does the current situation also bring any advantages? Every challenge results in new opportunities. Although the online congresses will most likely never fully replace real-life experience, it was found that many work meetings can be held more efficiently via online communication. Saving time, cutting costs of travel and accommodation, plus other expenses, may help to allocate limited resources where needed. Similarly, while practical medical education and training cannot be substituted for remote broadcasting, many theoretical presentations can. More importantly, epidemic of COVID-19 is a unique opportunity for mental health research, to study individual and population consequences of the virus, its impact on psychiatric patients. It is still early to predict whether and when research, training, meetings, and other academic activities return back to “normal”, but appears that some changes are here to stay.

**Disclosure:**

No significant relationships.

